# Trailing-Edge Flap Control for Mitigating Rotor Power Fluctuations of a Large-Scale Offshore Floating Wind Turbine under the Turbulent Wind Condition

**DOI:** 10.3390/e20090676

**Published:** 2018-09-06

**Authors:** Bofeng Xu, Junheng Feng, Tongguang Wang, Yue Yuan, Zhenzhou Zhao, Wei Zhong

**Affiliations:** 1 College of Energy and Electrical Engineering, Hohai University, Nanjing 211100, China; 2 Jiangsu Key Laboratory of Hi-Tech Research for Wind Turbine Design, Nanjing University of Aeronautics and Astronautics, Nanjing 210016, China

**Keywords:** trailing-edge flap, control strategy, floating wind turbine, turbulence, free vortex wake

## Abstract

A trailing-edge flap control strategy for mitigating rotor power fluctuations of a 5 MW offshore floating wind turbine is developed under turbulent wind inflow. The wind shear must be considered because of the large rotor diameter. The trailing-edge flap control strategy is based on the turbulent wind speed, the blade azimuth angle, and the platform motions. The rotor power is predicted using the free vortex wake method, coupled with the control strategy. The effect of the trailing-edge flap control on the rotor power is determined by a comparison with the rotor power of a turbine without a trailing-edge flap control. The optimal values of the three control factors are obtained. The results show that the trailing-edge flap control strategy is effective for improving the stability of the output rotor power of the floating wind turbine under the turbulent wind condition.

## 1. Introduction

Wind power has been developing rapidly worldwide due to fossil fuel energy depletion and environmental pollution. Offshore wind power is characterized by high wind energy density, high annual utilization hours, and close proximity to a power load center, and these advantages provide an important direction for future wind power development [1]. Although offshore wind power technology is relatively mature, the economy and reliability of wind turbines with fixed bases have decreased significantly with the expansion of wind turbine installation in deep-water areas and the associated advantages of a floating base. In 2009, the first spar-type full-scale floating offshore wind turbine, Hywind, was successfully installed and commissioned [2]. Subsequently, additional full-scale floating offshore wind turbines have been installed worldwide [3].

Ocean waves, ocean currents, strong winds, and the complex marine environment pose a series of problems to the normal operation of floating wind turbines. First, turbulent wind affects the inflow velocity and angle of the wind turbine blades. The inertia of large-scale wind blades is large and the individual pitch control is difficult to achieve, due to the rapidly changing aerodynamic loads under turbulent wind conditions [4]. Second, although the wind shear coefficient is smaller offshore than on land, the influence of the wind shear on the rotor power of the wind turbine cannot be ignored because the large hub height and long blades of offshore wind turbines cause a significant difference in the wind speed at the highest and lowest points of the wheel. Third, the floating platform swings periodically in a certain direction under the influence of the waves and currents. This phenomenon produces a large negative effect on the fatigue loads of the relevant components of the wind turbine [5]. The periodic motion of the floating foundation also causes periodic fluctuations in the rotor power, and affects the quality of the electric power output [1]. The 5 MW floating offshore wind turbine platform built by the US National Renewable Energy Laboratory (NREL) provides reliable basic data for offshore wind energy research [6]. Since then, research on load fluctuations and optimal control of floating wind turbines has also been conducted extensively in the industry [7]. Research on the use of blade flaps in wind turbines [8,9] has indicated that the trailing-edge flap structure of the wind turbine blade has technical advantages on the floating wind turbine. The wind turbine blade flaps do not only mitigate the load fluctuation effectively, but also supplement the traditional pitch control, which results in a more flexible and robust wind turbine control system.

Most of the aerodynamic analysis software packages such as Bladed and FAST are based on the Blade Element Momentum (BEM) theory. However, the wake-induced velocity in the BEM theory is the average induced velocity, and large errors can occur in the calculations, requiring many corrections. The vortex theory provides more accurate results for the wake flow field of the wind turbine when calculating the aerodynamic performance, and the induced velocity of the flow field is obtained directly from the wake flow [10]. Therefore, the vortex theory is suitable for calculating the unsteady aerodynamic performance of wind turbines. The free vortex wake (FVW) method is based on the vortex theory, and has been successfully applied to the aerodynamic performance calculation of wind turbines.

The main objective of this study is to propose a trailing-edge flap control strategy for a large-scale offshore floating wind turbine to mitigate the rotor power fluctuations in the turbulent wind condition. The NREL 5 MW floating wind turbine is used as an example for the calculations. The previously developed FVW model [10,11] is used to calculate the rotor power of the wind turbine with the proposed trailing-edge flap control.

In Section 2, the platform motions of the floating wind turbine are described. Section 3 describes the turbulent wind condition. Section 4 describes the blade structure with the trailing-edge flap and control strategy for mitigating the rotor power fluctuations. Section 5 briefly introduces the FVW method. The results are presented in Section 6, and they include the control effect on the rotor power under different unsteady conditions. The conclusions are drawn in Section 7.

## 2. Platform Motions of the Floating Wind Turbine

**Figure 1:**
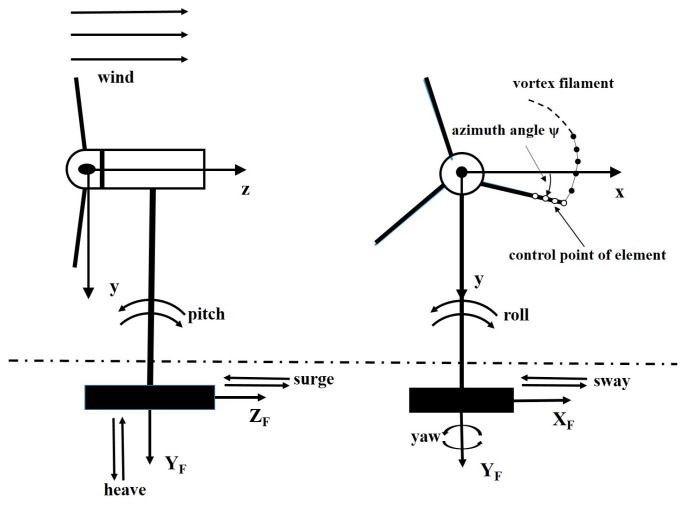
Coordinate system and the six degree of freedom motions.

As shown in Figure 1, the motion of the floating wind turbine platform can be described using a global coordinate system (*X*_F_, *Y*_F_, *Z*_F_) and a shaft coordinate system (*x*, *y*, *z*) originating at the shaft of the turbine. Assuming that the wind turbine is a rigid structure, the floating wind turbine platform has six degree of freedom, in terms of translational and rotational motions in the global coordinate system; these are surge, sway, heave, pitch, roll, and yaw.

**Figure 2:**
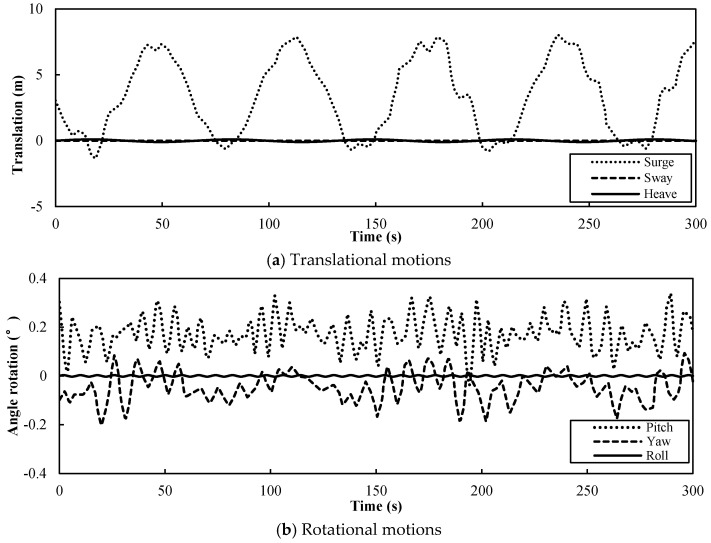
Time history of the tension leg platform (TLP) motions of the US National Renewable Energy Laboratory (NREL) 5 MW turbine for the rated operating condition.

The NREL 5 MW wind turbine is located on a floating tension leg platform (TLP). The rated operating condition is maintained at the rated speed of 11.4 m/s and the rotor speed is 12.1 rpm. The resulting FAST-simulated platform kinematics of the TLP [12] for the rated operating condition for a 300 s simulation was used in this study. Figure 2a shows the three translational motions, and Figure 2b shows the three rotational motions. It was observed that the mean and amplitude of the yaw, heave, and roll motions were small. The yaw motions had little effect on the wind turbine for a small angle, as the analysis of the influence of each degree-of-freedom motion on the aerodynamic performance showed. However, the pitch and surge motions had a large influence on the blade inflow of the wind turbine [12]. Therefore, in this study, we only analyzed the influence of the pitch and surge motions on the aerodynamic performance of the floating wind turbine.

## 3. Turbulence Wind Condition

**Figure 3:**
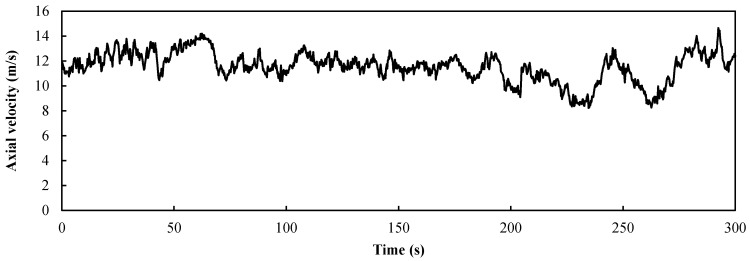
Axial velocity for the rated speed of 11.4 m/s in the turbulent condition.

In land-based wind farms or offshore wind farms, the main factor affecting the load fluctuations of wind turbines, is turbulent wind. Due to the lack of relevant measured data of offshore turbulent wind, we simulated the turbulence of the offshore wind field using a turbulence model. The wavelet transform is an appropriate method to detect the local similarity in time-series data of turbulence [13]. In this study, a one-dimensional velocity change (axial component) was considered at the hub center height of 90 m under the turbulent condition. The wavelet inverse transformation method [14] was used to calculate the turbulence wind field according to the advanced von Karman power density spectrum. The axial velocity varied around the rated wind speed of 11.4 m/s from 0 to 300 s, as shown in Figure 3. The roughness of the ocean surface was 0.001 mm, and the turbulence intensity was 0.0933.

**Figure 4:**
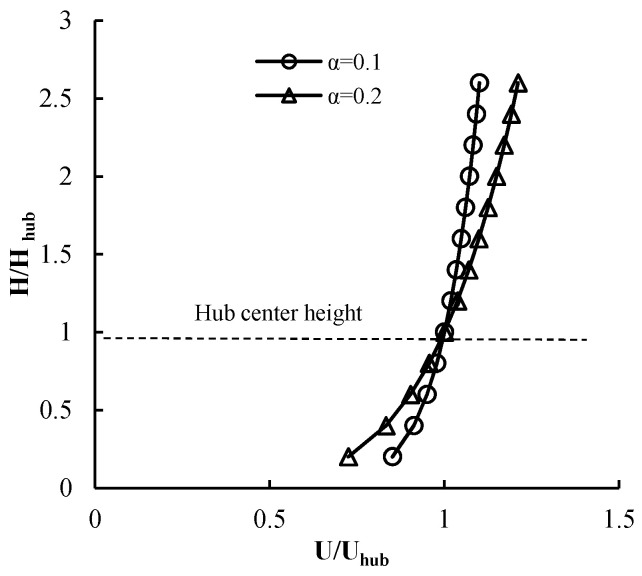
Atmospheric boundary layer profiles.

**Figure 5:**
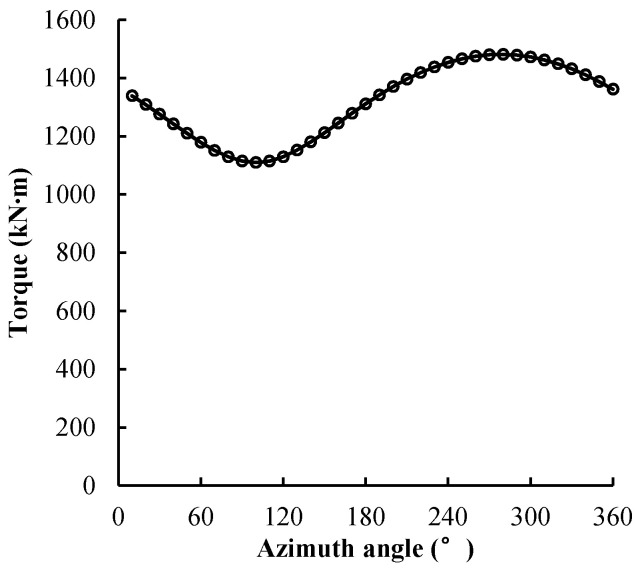
Aerodynamic torque of a single blade of the NREL 5 MW wind turbine during a rotation period under wind shear at the rated wind speed.

Wind shear exists in the atmosphere near the ground and sea surface because of the topography and the sea surface roughness. The oncoming boundary layer wind velocity profile is described as:

(1) $$U\left(h\right)=U_{ref}\cdot {\left(\frac{h}{h_{ref}}\right)}^{\alpha }$$

where *h_ref_* is the reference height (hub center height) and *U_ref_* is the wind speed at the reference height. The power law exponent *α* is associated with the local terrain roughness. Figure 4 shows the wind shear distribution near the ground, in which *α* = 0.1 is the value for the offshore sea and *α* = 0.2 is the value for land. Although the power law exponent is smaller offshore, the wind turbine output force changes due to the wind shear, because the large-scale offshore wind turbine has a diameter of more than 100 m. The hub center height of the NREL 5 MW wind turbine was 90 m, and the diameter of the rotor is 126 m. At a hub wind speed of 11.4 m/s, the maximum wind speed of the wheel reached 12.02 m/s, and the lowest wind speed is 10.11 m/s. Figure 5 shows the aerodynamic torque of a single blade of the NREL 5 MW wind turbine during a rotating period under wind shear at the rated wind speed. The range of the aerodynamic torque during the period is 1110–1482 k∙Nm. The amplitude is 28% of the average. Therefore, the wind shear needs to be taken into account.

## 4. The Trailing-Edge Flap Control Strategy

### 4.1. The Blade Structure with the Trailing-Edge Flap

**Figure 6:**
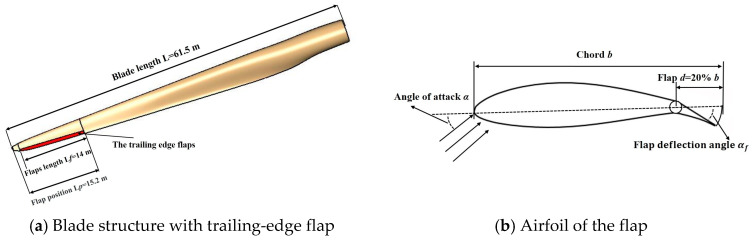
NREL 5 MW wind turbine blade with trailing-edge flap.

In this study, a trailing-edge flap structure for the wind turbine was proposed. The trailing-edge flap structure used in the NREL 5 MW wind turbine blade was based on the results of a study by Zhang et al. [15] on the optimization of the structural parameters of the trailing-edge flap of wind turbines. As shown in Figure 6a, the blade length was 61.5 m and the radial flap length of the red portion was 14 m along the axis direction of the blade; the flap was located at 1.2 m from the tip of the blade, and extended to a distance of 15.2 m from the tip. The relative thickness of the airfoil with the flaps was changed to 18%, which was convenient for installation and calculation purposes. The flap length comprised 20% of the length of the chord, as shown in Figure 6b.

### 4.2. The Aerodynamic Performance of the Trailing-Edge Flap

**Figure 7:**
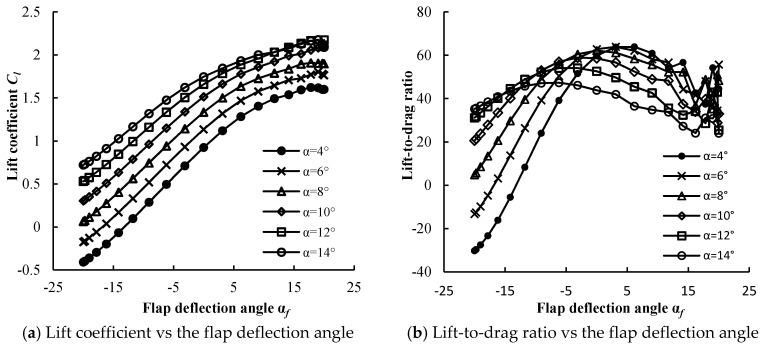
Aerodynamic performance of the airfoil with a trailing-edge flap.

The control system adjusts the torque coefficient and the thrust coefficient of the blades, to mitigate the load fluctuations of the blade through the deflection control of the flap. Therefore, the aerodynamic performance of the airfoil with the flaps is vital. Figure 7 shows the lift and drag coefficient data of the airfoil with different flap deflection angles. In this study, the NREL 5 MW wind turbine was simulated under the condition of the rated wind speed. When the NREL 5 MW wind turbine ran at the rated wind speed of 11.4 m/s, the blade pitch angle was 0° and the rotational speed was 12.1 rpm. The angle of attack at the blade tip was about 8°, and the lift and drag coefficient data, in the range of 4–14°, are given in Figure 7. It can be seen that the lift coefficient increased with the increase in the flap deflection angle for the same attack angle, and the lift-to-drag ratio increased first and then decreased. It is worth noting that the smaller the attack angle, the faster the lift-to-drag ratio increased, and the larger the adjustable range became. Therefore, a smaller attack angle of the blade is preferred. In the range of the flap deflection angle of 10~15°, the lift-to-drag ratio decreased, whereas the lift-to-drag ratio was unstable in the range of 15~20°. A comprehensive analysis indicated that *α_f_* = −5° was optimal for the origin of the flap, and −20~10° was the optimum range of the flap deflection angle. 

### 4.3. Control Strategy

The flap control method is also important and determines the effectiveness of the flap control. A simple flap control method based on wind speed, blade azimuth angle, and platform motion was proposed to verify the effectiveness of the flap control. More efficient and practical control methods require further study.

The control strategy under the turbulent wind was based on the two average wind speeds (*U* and *U_t_*). It was assumed that the instantaneous wind speed was measured four times per second by the nacelle anemometer. The control scheme is defined as:

(2) $$\alpha_{ft}(t)=a\cdot (U-U_{t})$$

where *a* is the control factor for the turbulence and *U* is the average wind speed over 60 s. In order to avoid the frequent change of the flap deflection angle, *U_t_* is defined as the average wind speed over one second, and is expressed in Equation (3):

(3) $$U_{t}=\frac{u_{t}+u_{t-0.25}+u_{t-0.5}+u_{t-0.75}+u_{t-1}}{5}$$

The effect of the wind shear on the wind turbine is related to the position of the blade. The maximum load is obtained at the highest point of the turbine wheel and the minimum load is obtained at the lowest point. The aerodynamic loads of the turbine are determined by the superposition of three blades and theoretically, there are three peaks and three lows in one cycle. The proposed control scheme controls the three blades to mitigate the load fluctuations of the wind turbine according to the azimuth angle of the blade, which can be described as:

(4) $$\alpha_{fs}(t)=b\cdot (\frac{U_{hub}-U_{tip}}{\left|U_{hub}-U_{\max}\right|})\cdot \left|\frac{U_{hub}-U_{tip}}{U_{hub}-U_{\max}}\right|$$

where *b* is the flap control factor of the wind shear. *U_hub_* is the wind speed at the hub and *U_tip_* is the wind speed at the blade tip, which is calculated by Equation (1) with *α* = 0.1 and it can described as:

(5) $$U_{tip}=U_{hub}{\left(\frac{h_{hub}-R\sin\psi }{h_{hub}}\right)}^{\alpha }$$

where *h_hub_* is the height of the hub, *R* is the radius of the turbine wheel, and *ψ* is the blade azimuth angle. *U*_max_ is the maximum value of *U_tip_*.

For the control of the pitch and surge motions, the deflection angle *α_p_*(*t*), and the angular velocity *ω_p_*(*t*) of the pitch motion, the velocity *v*(*t*) of the surge motion, and the azimuth angle of the blade *ψ* have to be measured. Equation (6) describes the flap control method for the platform motion:

(6) $$\alpha_{fp}(t)=c\cdot \left(\omega_{p}(t)\cdot (h_{hub}-R\sin\psi )+v(t)\cdot \cos\alpha_{p}(t)\right)+(1-\cos\alpha_{p}(t))$$

where *c* is the control factor for the platform motion. In this scheme, the position of the flaps were determined by calculating the wind velocity component of the blade inflow, caused by the platform motion.

$\alpha_{ft}$, $\alpha_{fs}$, and $\alpha_{fp}$ are the flap deflection angles due to wind speed change, blade azimuth angle, and platform motion. A linear superposition method was used to determine the position of the flaps under multi-input conditions. The flap deflection angle is defined as:

(7) $$\alpha_{f}=\{\begin{array}{c} -20\\ \alpha_{ft}(t)+\alpha_{fs}(t)+\alpha_{fp}(t)-5\\ 10 \end{array}\begin{array}{c} ,\\ ,\\ , \end{array}\begin{array}{c} \alpha_{ft}(t)+\alpha_{fs}(t)+\alpha_{fp}(t)<-15\\ -15\leq \alpha_{ft}(t)+\alpha_{fs}(t)+\alpha_{fp}(t)\leq 15\\ \alpha_{ft}(t)+\alpha_{fs}(t)+\alpha_{fp}(t)>15 \end{array}\;$$

## 5. Description of the FVW Model

The FVW model assumes that the flow field is incompressible and potential. The blade is modeled by a Weissinger-L model [16] as a series of straight constant strength vortex segments lying along the blade quarter chord line. The control points are located at a 3/4-chord at the center of each panel. The wake vortices extend downstream from the 1/4-chord, forming a series of horseshoe filaments. The trailing filaments cut off at a wake age angle of 60° in the near-wake and roll up and form a single tip vortex filament in the far-wake. The strength of the tip vortex equals the global maximum bound vorticity over the span of the blade. The release point of the tip vortex is the tip of the blade. The detailed calculation process of the FVW model can be found in [10]. The validation of the FVW model on blade airload predictions of offshore floating wind turbines is also presented in Ref. [10].

## 6. Results and Discussion

Appropriate flap control parameters are required to achieve a good control effect. In the following section, we discussed the influence of the control parameters on the control performance under the unsteady conditions comprised of turbulent wind, wind shear, and platform motion.

**Figure 8:**
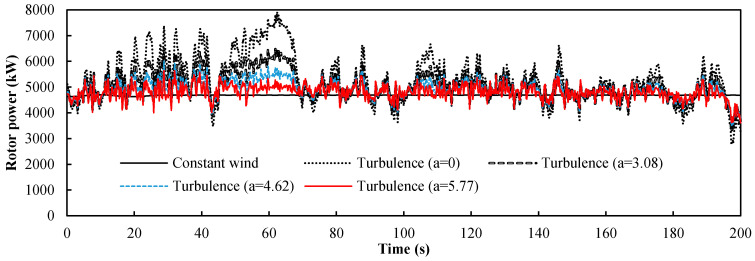
Power response of the floating wind turbine under turbulent wind from 0 to 200 s.

We set *a* as the ratio of the flap control angle ∆*α_ft_* for the turbulence to the maximum deviation value ∆*u_t_* of the turbulent wind (*a* = ∆*α_ft_*/∆*u_t_*). It can be seen from Figure 3 that the maximum deviation ∆*u_t_* of the turbulent wind was 2.6 m/s, and the power curves of the four control factors are obtained by setting ∆*α_ft_* as 0°, 8°, 12°, and 15° respectively. The power response of the wind turbine from 0 to 200 s is shown in Figure 8 and was based on the turbulent wind condition shown in Figure 3. The input of the control group is a constant wind and the rotor power of the wind turbine is 4.7 MW, slightly less than the rated power. This is because the origin of the flap was −5°. It was obvious that the turbulent wind had a large influence on the stability of the wind turbine and the maximum deviation of the power fluctuation reaches 60% of the stable power when *a* = 0. We present four curves with different control factors. The amplitude of the curve for the turbine with the controlled flap (*a* > 0) was clearly smaller than that of the turbine with the fixed flap (*a* = 0). In Table 1, the statistical results of the rotor power with the four different control factor values of *a* are summarized. It is evident that the average value varied little, but the larger the value *a*, the smaller the standard deviation was, and the closer the maximum and minimum were to the mean value. This shows that the larger the value of *a*, the better the flap control system worked. Therefore, the maximum value of *a* (5.77) was the appropriate value for this turbulent condition.

**Figure 9:**
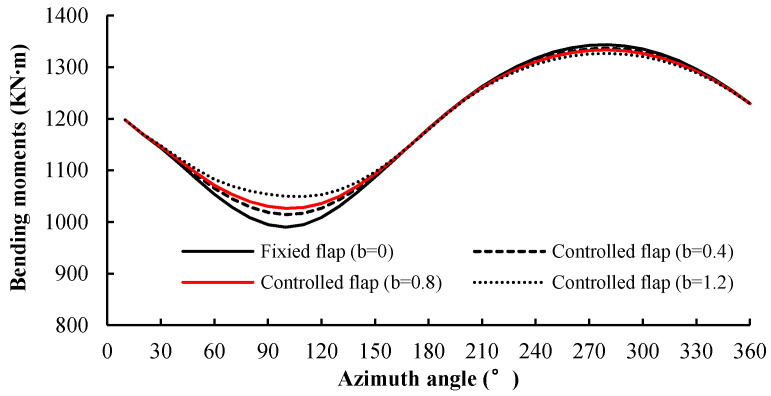
Blade root aerodynamic bending moments of a single blade during a rotation period under shear wind.

**Figure 10:**
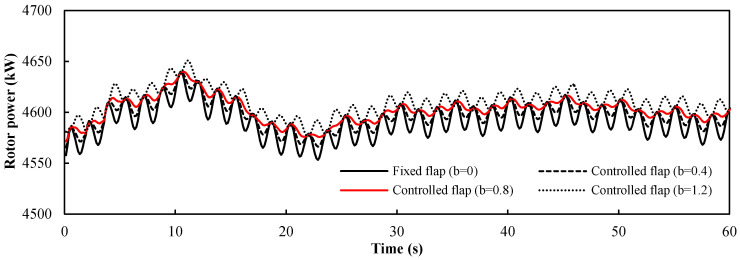
Power response of the floating wind turbine under wind shear from 0 to 60 s.

The control factor *b* for the wind shear was equal to the maximum flap deflection angle ∆*α_fs_*, which was set as 0°, 0.4°, 0.8°, and 1.2°. It can be seen from Figure 9 that the wind shear could cause obvious aerodynamic bending moment fluctuations at the root of a single blade. The control effect on the aerodynamic bending moment at the 90° azimuth angle was more obvious than that at the 270° azimuth angle, because of the different wind speed gradient. However, due to the superposition of the three blades, the total aerodynamic power of the turbine did not fluctuate as much, as shown in Figure 10. Under the wind shear condition, the rotor power curve exhibits low-frequency fluctuations in the first 30 s. This occurs because the flow field was calculated by the constant flow field prior to time 0, and the wind shear field was calculated after time 0, and the induction of the new wake had a continuous effect on the blade load. In the four curves, the amplitude decreased as the value of *b* increased, but when *b* = 1.2, the amplitude of the power fluctuation reversed. The statistical metrics of the four values of the control factor *b* are shown in Table 2. The mean value of each group of data was very close, and the maximum and minimum values were very similar. The change values between the maximum and minimum values were about 70 kW. At *b* = 0.8, the standard deviation reached the minimum value; therefore, 0.8 was the appropriate value of the control factor *b*.

**Figure 11:**
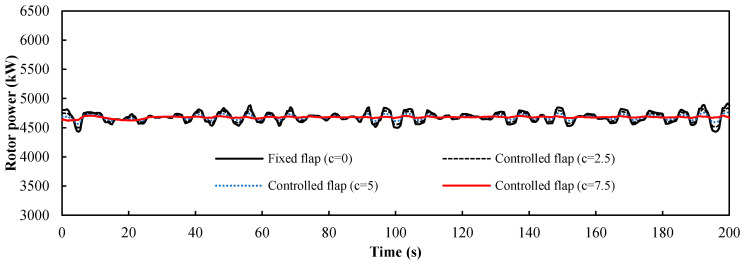
The power response of the floating wind turbine under pitch motions from 0 to 200 s.

**Figure 12:**
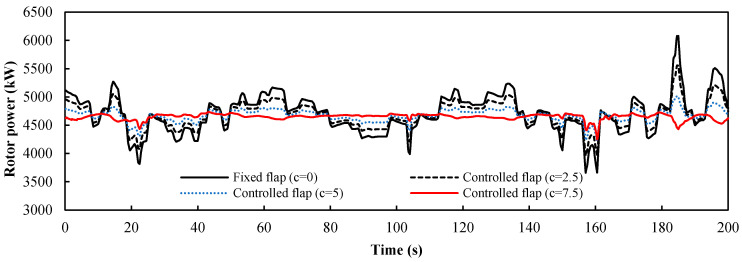
Power response of the floating wind turbine under surge motions from 0 to 200 s.

**Figure 13:**
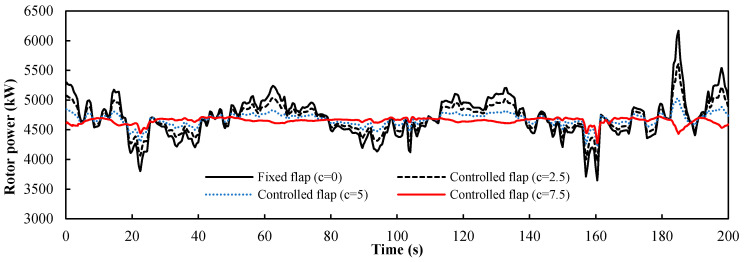
Power response of the floating wind turbine under pitch and surge motions from 0 to 200 s.

The analysis of the platform motions indicates that the influence on the wind turbine load mainly consists of pitch and surge motion. The control factor *c* is set as the ratio of the maximum flap deflection angle ∆*α_fp_* to the maximum deviation ∆*u_p_* of the axial wind velocity caused by two kinds of platform motions (*c* = ∆*α_fp_*/∆*u_p_*). Here ∆*u_p_* = 2 m/s can be obtained from the platform motion data. Four values were selected to study the influence of the control factor on the control performance. Figure 11 is the load response of the pitch motions, and Figure 12 is the load response of the surge motions. It was evident that the power fluctuation caused by the surge motions was larger than that caused by the pitch motions for the same flap deflection angle. The frequency of the load fluctuation caused by the platform was smaller than that caused by the turbulent wind and the greater the control factor, the better the control effect is. Figure 13 shows the load response of the wind turbine under the combined pitch and surge motions. The result is similar to the load response of the surge motion, which indicates that the surge motion is the dominant motion type. There are no apparent fluctuations when the control factor *c* is equal to the maximum value 7.5, which shows that the trailing-edge flaps have a positive effect on the low-frequency fluctuations, such as the platform motion. The statistical metrics of the curves with different control factor value *c* are shown in Table 3. As *c* increases, the mean value decreases only slightly but the standard deviation decreases markedly. When *c* reaches the maximum value, the standard deviation reaches the minimum value. As a result, 7.5 is the optimal value of the control factor *c*.

**Figure 14:**
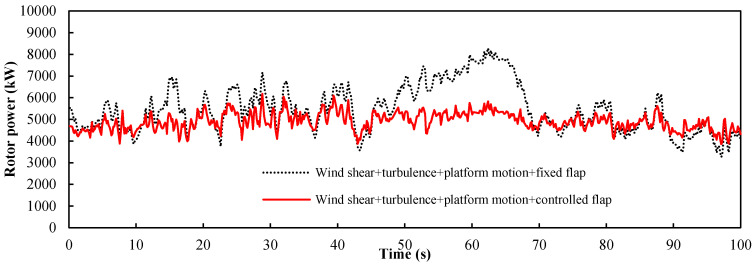
Power response of the NREL 5 MW floating wind turbine under the turbulent wind condition.

When the input wind condition is the turbulent wind, turbulent wind, wind shear and platform motions will affect the rotor power together. The above results indicated that the trailing-edge flap had a good mitigation effect on the power fluctuations caused by the three kinds of dynamic inputs when *a* = 5.77, *b* = 0.8, and *c* = 7.5. We used these values for the three parameters to achieve the linear control of the flap. Figure 14 shows the power response of the turbine for all three dynamic inputs. Table 4 shows the statistical metrics of the two curves in Figure 14. It was observed that the controlled trailing-edge flap mitigates the rotor power fluctuations of the wind turbine under these conditions. Under the combined action of the three kinds of dynamic inputs, the controlled flap reduced the standard deviation of the power fluctuations from 1095 to 404.

## 7. Conclusions

In this study, a trailing-edge flap control strategy is integrated into the blade of the NREL 5 MW wind turbine to cope with the turbulent wind, wind shear, and the motions of the floating platform. The rotor power of the wind turbine is calculated using the FVW method. A simple flap control method based on wind speed, blade azimuth angle, and platform motion is proposed to achieve effective flap control. The results show that turbulent wind has the largest impact on the stability of the floating wind turbine. The optimal values of three control factors for turbulent wind, wind shear, and platform motions are obtained. The controlled trailing-edge flap with appropriate control factors effectively mitigates the power fluctuations caused by turbulent wind, wind shear, and platform motion. The proposed trailing-edge flap control strategy need to be validated by some experimental studies in the future, and then it can be applied in the large-scale offshore floating wind turbine.

## Figures and Tables

**Table 1:** The statistical metrics of the rotor power with different control factor values *a*.

*a*	0	3.08	4.62	5.77
Mean value (kW)	5295	5050	4913	4807
Standard deviation (kW)	905	533	365	279
Maximum (kW)	7921	6493	6007	5640
Minimum (kW)	2763	3290	3501	3615

**Table 2:** The statistical metrics of the rotor power curves with different control factor values *b*.

*b*	0	0.4	0.8	1.2
Mean value (kW)	4592	4598	4602	4609
Standard deviation (kW)	16.1	13.6	12.8	13.7
Maximum (kW)	4639	4639	4640	4651
Minimum (kW)	4553	4565	4571	4576

**Table 3:** The statistical metrics of the curves with different control factors for the pitch and surge motions.

*c*	0	2.5	5	7.5
Mean value (kW)	4702	4691	4675	4650
Standard deviation (kW)	333	218	105	52
Maximum (kW)	6166	5609	5025	4721
Minimum (kW)	3646	3967	4220	4272

**Table 4:** Statistical metrics of the two curves in Figure 14.

Input Conditions	Wind Shear, Turbulence, Platform Motion, Fixed Flap	Wind Shear, Turbulence, Platform Motion, Controlled Flap
Mean value (kW)	5477	4902
Standard deviation (kW)	1095	404
Maximum (kW)	8283	6176
Minimum (kW)	3266	3857
